# Active inhibition of the retro-cue effect in visual working memory: Evidence from event-related potential

**DOI:** 10.1177/20416695231182290

**Published:** 2023-06-27

**Authors:** Chao Gao, Qi Zhang, Xiaoxiao Zhang

**Affiliations:** School of Psychology, 66523 Liaoning Normal University, Dalian, China; School of Psychology, 47890 Shenzhen University, Shenzhen, China

**Keywords:** retro-cue effect, visual working memory, internal selective attention, active inhibition, event-related potential

## Abstract

This study used the event-related potential (ERP) technique to investigate whether active inhibition exists in retro-cue Effect (RCE) in visual working memory using modified retro-cue tasks. In this modified task, the participants were first asked to memorize six color blocks and then presented with directed remembering or directed forgetting cues; finally, their working memory performance was tested. For behavioral results, due to the extension of the memory interval, this study did not find RCE in accuracy but reflected it in the total reaction time. For ERP results, the frontal late positive potential (LPP) followed by the directed forgetting condition was larger than that followed by directed remembering and baseline conditions, and there was no significant difference between directed remembering and baseline conditions. There was no significant difference in parietal P3 followed by both the directed remembering and directed forgetting conditions, which were significantly larger than the baseline condition. This result reveals that active inhibition plays an important role in directed forgetting RCE. There was a correlation between parietal P3 and frontal LPP with the same time window but different scalp regions in the directed forgetting condition, indicating a potential relationship between active inhibition and retelling in directed forgetting RCE.

Visual working memory is responsible for the temporary storage and processing of visual information and is an important part of the human cognitive system (Hollingworth et al., 2008; Luck & Vogel, 2013; Ma et al., 2014). However, the capacity of visual working memory is limited: it can only represent limited input information, and these representations are selected and controlled by attention. Depending on the goal, attention can include external attention, which is gathered from sensory information, and internal attention, which in turn relies on mental representations (Chun et al., 2011). Among them, the influence of internal attention on visual working memory has attracted increasing attention from researchers (Awh et al., 2007; Liang et al., 2018; Luck & Vogel, 1997; Ye et al., 2014; Ye et al., 2020; Zhang & Luck, 2008). Griffin and Nobre (2003) added a retro-cue to the change-detection paradigm to investigate the effect of internal attention on visual working memory representations. Specifically, the participants were first asked to remember a series of items and then presented with cues indicating task-relevant information. Finally, the researchers measured the effect of cues on item recall. The results showed that compared with trials without cues, trials with cues showed better task performance. This phenomenon has been named the retro-cue effect (RCE) and has also been identified by other researchers (Matsukura & Vecera, 2015; Schneider et al., 2015).

As retro-cues are important for improving visual working memory task performance, researchers have examined the factors that influence RCE (Fan et al., 2021; Fu et al., 2022; Lin et al., 2021; Lin & Guo et al., 2023; Luo et al., 2023). For example, Williams and Woodman (2012) found that cue validity influenced RCE, similar to Fu et al. (2021). Lin et al. (2021) argued that RCE is closely related to the selection of attention with limited resources. Fan et al. (2021) ascertained that the perceptual salience of the stimulus also influenced RCE. More recently, Lin and Guo (2023) discovered that sustained attention plays an important role in RCE, while Luo et al. (2023) stated that whether information in visual working memory completes consolidation is a key factor in RCE. In summary, researchers have examined the factors that influence RCE; however, as important as these factors are, the mechanisms of RCE need to be elucidated further.

The mechanism of RCE can be explained by the continuous resource model and the boost and bounce model. The former posits that visual working memory resources are limited and that internal attention can selectively allocate them to each representation in visual working memory (Just & Carpenter, 1992). Therefore, task-relevant information will receive more resources, and the memory recall will be enhanced, while task-irrelevant information will receive fewer resources, and the memory recall will be reduced, resulting in RCE. This model has been supported by several experimental studies, such as Williams and Woodman's (2012) research that posited that limited internal attention resources are selectively refocused on task-relevant information to enhance task-relevant recall. Ye et al. (2020) asserted that retro-cues focus internal attentional resources on task-relevant information, which is the cause of RCE.

The boost and bounce model proposes that working memory is a workspace in which task-relevant information is enhanced due to excitatory manipulation, but task-irrelevant information is weakened due to inhibitory operations (Olivers & Meeter, 2008). This model has also been supported by experimental research. For example, Poch et al. (2018) and Schneider et al. (2019) discovered that visual working memory not only enhances task-relevant information but also suppresses task-irrelevant information. In summary, the continuous resource model considers improved retention of task-relevant information an active process while weakening task-irrelevant information is due to passive reduction. The boost and bounce model proposes that both the enhancement of task-irrelevant information and the weakening of task-irrelevant information are active processes. Although both models can explain RCE, a conflict point exists; whether the weakening of task-irrelevant information is active inhibition or passive elimination and which model is more practical in explaining task-irrelevant information representation has not been explored by experiments.

It is impossible to report which model is more practical by behavior research alone because different cognitive processes may produce similar behavior results. Event-related potential (ERP) technology can help us understand the neural activity pattern behind the behavior. Previous studies used contralateral delayed activity (CDA), a neural indicator that reflects visual working memory load, and found that retro-cues reduced visual working memory load through selective attention to improve task performance (Murray et al., 2009; Williams & Woodman, 2012). The CDA began to appear approximately 300 ms after the cue was presented and lasted until the memory item was detected, which made it difficult to observe the reason for the decrease in load in this neural indicator. Subsequent research using the α-band indicator of neural oscillation lateralization found that cues would induce an α-band energy response, representing active inhibition (Poch et al., 2018; Schneider et al., 2019). However, the psychological meaning of the α-band is controversial. The lateralized α indicator may reflect the distribution path of internal selective attention rather than the enhancement or inhibition of working memory representations (Gunseli et al., 2018; Poch et al., 2018; Schneider et al., 2017). Therefore, the presence of an active inhibitory component in RCE remains controversial. More evidence is needed to determine whether active inhibition exists.

The P3 component is a positive wave appearing in the 400–600 ms, representing the rehearsal of information and the degree of cognitive resources occupied thereby (Azizian & Polich, 2007; Christie et al., 2012; Gilmore et al., 2018; Patrick et al., 2015; Polich, 2007). Therefore, the enhancement of task-relevant information may be reflected in the P3 component of the central-parietal lobe. Recently, some studies have reported positive slow waves in the frontal lobe (frontal late positive potential [LPP]), whose time window is similar to that of P3 (Cano & Knight, 2016; Ma et al., 2020). Compared with remember conditions, the amplitude of the frontal LPP was larger under inhibition conditions, and researchers believe that this may reflect the increased activity of the dorsolateral prefrontal cortex (DLPFC) during the inhibition process, realizing the continuous inhibition of task-irrelevant information. Some studies have found that the cognitive process of intentional control (the emotional reappraisal of intentional control) induces greater frontal LPP amplitudes than neutral conditions (Bernat et al., 2011; Moser et al., 2014; Shafir & Sheppes, 2015). Therefore, frontal LPP is considered a neural indicator of cognitive effort. The increase in frontal LPP amplitude is closely related to the increase of “motivational attention,” which is extremely sensitive to top-down cognitive processes such as inhibition control. Some studies on the influence of directional attention on frontal LPP amplitudes support this view (Dunning & Hajcak, 2009; Hajcak et al., 2009). Kan et al. (2021) also found that the frontal LPP amplitude of the continuous inhibition task was larger than that of the neutral task, indicating that the continuous inhibition task may be more reflected in the LPP component than the “all or nothing” inhibition task is reflected in N2. Therefore, we expect the weakening of task-irrelevant information to be reflected in the frontal LPP.

To investigate the active inhibition process induced by retro-cues more accurately, this study modified the retro-cue task of visual working memory and recorded ERP data during the experiment. In the modified paradigm, the interval between memory and cues was set as 800–1200 ms random time to avoid the preparation state caused by participants’ familiarity with the memory stage and cue stage intervals. To directly compare the neural activities of the two cues in the time course, the two types of cues were set as a within-subject variable. This is different from the research by Williams and Woodman (2012).

This study mainly focused on examining whether active inhibition exists in RCE using a modified visual working memory retro-cue task, that is, whether the weakening of task-irrelevant information is due to active inhibition or passive abatement. If the weakening of task-irrelevant information is due to active inhibition, cues should also induce significantly enhanced frontal LPP in addition to the P3 component reflecting task-relevant information enhancement. By contrast, if the weakening of task-irrelevant information is due to passive subtraction, the cue does not induce significantly enhanced frontal LPP.

## Methods

### Participants

Twenty-five participants participated in this experiment and their mean age was 21.80 ± 2.062 years. All participants had normal or corrected vision, without color blindness or color weakness. They were also right-handed with no psychiatric history or brain injuries. With the consent of the Academic Ethics Committee of Liaoning Normal University, the participants gave informed consent and received payment after the experiment.

### Materials

In this study, seven different color blocks were used as memory materials: red (RGB: 255, 0, 0), purple (RGB: 237, 4, 140), green (RGB: 0, 165, 87), black (RGB: 0, 0, 0), yellow (RGB: 255, 255, 0), and white (RGB: 255, 255, 255), with a size of 1.25° × 1.25°. The fixation point divided the field of vision into left and right sections. Each visual field included three color blocks. The minimum distance between color blocks was 0.55°. The fixation point was 1.8° from the memory material in both visual fields. The directed remembering cue was a solid English word, “right/left,” while the directed forgetting cue was a hollow English word, “right/left,” with a size of 1° × 1.4°. All materials were presented on a gray background (RGB: 128, 128, and 128). All stimuli were presented using a Dell 27-inch 60 Hz display. The experimental program was written and run using E-Prime 2.0. The experiment was conducted in a dark, soundproof room, and the participants were placed approximately 60 cm from the display screen.

### Procedure

The experiment was divided into three conditions: directed remembering, directed forgetting, and baseline conditions.

In the directed remembering condition, the participants were first presented with a 500 ms fixation point and then presented with a 500 ms memory stage. After the 800–1200 ms memory interval, the participants were presented with a cue for 1000 ms, followed by another 1000 ms memory interval, and finally presented with a 5000 ms test stage (disappeared after pressing the key). If the cue was “right,” there were three items in the right field of vision during the test phase. Participants were required to judge whether the items in the right visual field were the same as those in the memory phase and the F key for “Yes” or J for “No.”

In the directed forgetting condition, the cues presented to participants were directed forgetting cues. For example, if the cues were “left,” the test stage presented three items of the right visual field, and the participants were to judge whether the items in the right visual field were the same as that in the memory stage. The other settings were the same as the directed remembering condition.

In the baseline condition, the cue was replaced by fixation, and the test phase randomly tested one side of the visual field.

**Figure 1.**
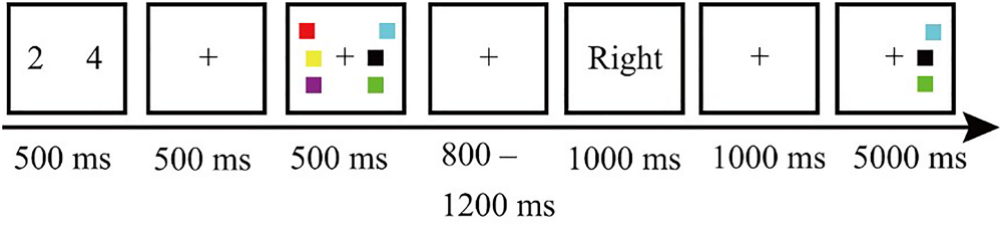
Example flowchart of the experiment. Each trial started with the voice inhibition task, followed by the modified retro-cue task of visual working memory. During the experiment, the participants were asked to repeat these numbers verbally.

Before each trial, participants were required to perform a voice inhibition task for 500 ms. Specifically, two numbers were randomly presented from 1 to 9 on both sides of the central fixation point (4° apart). The participants were required to repeat these two numbers verbally until the test stage was completed. Each condition had 120 trials and was randomly presented, with 360 trials divided into five blocks. Before the formal experiment, participants performed 12 practice experiments to familiarize themselves with the experiment. An example is shown in Figure 1.

### Electrophysiological Data Recording and Analysis

The EEG was recorded from 64 Ag/AgCl electrodes mounted in elastic caps, and the reference electrode was placed at the FCz site (Brain Products Inc., Germany). The electrode position was based on the extended 10–20 system, using a vertical facial electrode (under the left eye) and a horizontal facial electrode (to the right of the right eye) to record an electrooculogram (EOG). The EEG data were sampled at 500 Hz/channel with a 0.01–100 Hz band-pass, and before the recording, all electrode impedances were maintained below 5 kΩ. EEGLAB and ERPLAB were used for preprocessing. First, the EEG data were re-referenced as the average values at the left and right mastoids. After ocular correction (using independent component analysis), the data were filtered with a 0.1 Hz IIR, 50-Hz notch, and 30-Hz FIR filters. Trials contaminated with electrooculogram artifacts or those with artifacts due to amplifier clipping, bursts of electromyographic activity, or peak-to-peak deflections exceeding ±80 μV were excluded from averaging. The EEGs were averaged separately for each participant, and only trials with correct responses were included in the ERP averages. The range of valid trials for the directed remembering, directed forgetting, and baseline conditions were 62–104, 59–99, and 66–105, respectively. The voltage of the EOG electrode did not exceed 3 μV: specifically, for the directed remembering condition: −0.33 ± 0.42 μV, for the directed forgetting condition: 1.05 ± 0.52 μV, and for the baseline condition: −0.18 ± 0.50 μV.

The frontal lobe was divided into three scalp positions: left-frontal (F3 and FC3), middle-frontal (Fz and FCz), and right-frontal (F4 and FC4). The parietal lobe was also divided into left-parietal (CP3 and P3), middle-parietal (CPz and Pz), and right-parietal (CP4 and P4) scalp regions to reflect their EEG activities. According to previous studies, the N1 component reflects the early alert state of attention and is related to the response induced by the stimuli's visual and physical characteristics (Qi & Gao, 2020; Sponheim et al., 2006). This study used two types of cues: hollow for directed forgetting cues and solid for directed remembering cues. Therefore, N1 (100–180 ms) was selected for the analysis to determine whether the two types of cues evoked the same visual response. The P2 component is closely related to the selective attention process prior to cognitive control (Bergstrom et al., 2007; Lenartowicz et al., 2014; Mecklinger et al., 2009). Therefore, this study selected the P2 component (200–300 ms) to reflect the selective attention process before cognitive control. Based on this hypothesis, we selected the frontal LPP (400–750 ms) and occipital P3 to reflect the weakening of task-irrelevant information and the enhancement of task-relevant information.

SPSS was used for statistical tests, and the Greenhouse-Geisser correction was conducted for the effect of nonconformance with the spherical test. In addition, Bonferroni correction was performed for the main effect, interaction, postanalysis, and simple effect tests. The BF_01_ index was used for the Bayesian analysis of the critical *p-*value.

## Results

### Behavioral Results

The percentage of correct responses was taken as the accuracy index and was analyzed by one-way ANOVA at three levels (task type: directed remembering condition, directed forgetting condition, and baseline condition). The results showed that the accuracy of the three different conditions, directed remembering, directed forgetting, and baseline, were 78%, 75%, and 76%, respectively. The main effect of the task type was not significant, *F*(2, 48) = 2.431, *p* = .099.

The average reaction time of the correct trial was considered the indicator of the total reaction time (RT), and a one-way ANOVA at three levels (task type: directed remembering condition, directed forgetting condition, and baseline condition) was performed. The results showed that the RT of directed remembering, directed forgetting, and baseline were 1082, 1126, and 1265 ms, respectively. The main effect of task type was significant, *F*(2, 48) = 24.772, *p* < .001, *η_p_*^2^ = .508. The results of the simple effect test showed that the RT of the directed remembering condition was significantly lower than that of the directed forgetting and baseline conditions (*p*s < .006). The RT of the directed forgetting condition was significantly lower than that of the baseline condition (*p* = .001).

**Figure 2.**
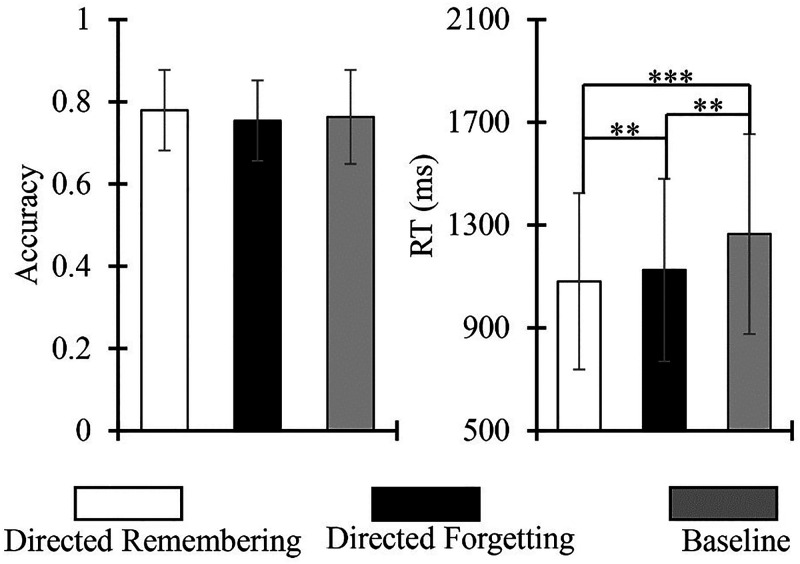
Accuracy index (left) and reaction time (right) of task types. Results of a one-way ANOVA revealed that task type (directed remembering, directed forgetting, and baseline) did not have a significant effect on the accuracy index. Task type had a significant effect on RT. The directed remembering condition had lower RT than directed forgetting and baseline conditions. Directed forgetting had significantly lower RT than baseline conditions. Bars represent standard error and asterisks denote significance (**p < .001; ***p < .001).

Abbreviations: ANOVA = analysis of variance; RT: reaction time.

Afterward, outcome measures (RT and ACC) were combined into the balance integration score (BIS), a standardized performance index (Liesefeld et al., 2015; Liesefeld & Janczyk, 2019, 2022). The BIS accounts for speed–accuracy (RT, ACC) trade-off by assigning equal weights to each measure. As higher ACC and lower RT scores indicate better performance, a higher BIS represents a better overall task performance. For BIS, the Friedman Test at three levels (task type: directed remembering condition, directed forgetting condition, and baseline condition) was performed. The results showed that the BIS for the three different conditions, directed remembering, directed forgetting, and baseline, were .065, .067, and .022, respectively. The main effect of the task type was not significant, χ^2^(2) = .750, *p* = .687 (Figure 2).

### ERP Results

A 3 (task type: directed remembering, directed forgetting, baseline) × 3 (frontal scalp regions: left frontal, middle frontal, right frontal) repeated-measures ANOVA was performed for N1 (100–180 ms), P2 (200–300 ms), and frontal LPP (400–750 ms).

During the N1 (100–180 ms) time window, the mean amplitudes of directed remembering, directed forgetting, and baseline conditions were −.796, −.568, and −0.21 μV, respectively. The main effect of task type was significant, *F*(2, 48) = 5.180, *p* = .009, *η_p_*^2^ = .178. The post-hoc paired test results showed that the N1 amplitude of the directed remembering condition was significantly larger than that of the baseline condition (*p* = .033). There was no significant difference between the directed remembering and directed forgetting conditions (*p* = .718), as with the directed forgetting and baseline conditions (*p* = .145). The main effect of the frontal scalp region was not significant, *F*(2, 48) = .698, *p* = .502. Similarly, the task type × frontal scalp region interaction was not significant, *F*(4, 96) = 1.228, *p* = .304.

During the P2 (200–300 ms) time window, the mean amplitude of directed remembering, directed forgetting, and baseline conditions were 4.286, 4.940, and −.173 μV, respectively. The main effect of task type was significant, *F*(2, 48) = 48.529, *p* < .001, *η_p_*^2^ = .669, and the main effect of the frontal scalp region was significant, *F*(2, 48) = 12.243, *p* < .001, *η_p_*^2^ = .338. The interaction of task type × frontal scalp region was significant, *F*(4, 96) = 9.800, *p* < .001, *η_p_*^2^ = .290. A simple effect test showed that in the left and right frontal regions, the P2 amplitude of the directed remembering condition was significantly smaller than that of the directed forgetting condition (*p*s ≤ .034); however, there was no significant difference between the two conditions in the middle frontal region, *p* = .099. In the three regions of the frontal scalp, the amplitude of the directed forgetting condition was significantly larger than that of the baseline condition, *p*s ≤ .001. Similarly, the amplitude of the directed remembering condition was also significantly larger than that of the baseline condition in the three regions of the frontal scalp (*p*s ≤ .001). In the directed remembering condition, the amplitude of the middle frontal region was significantly larger than that of the left and right frontal regions (*p*s ≤ .001), but there was no significant difference between the left and right frontal regions (*p* = 1.000). In the directed forgetting condition, we also found that the middle frontal lobe was significantly larger than the left and right frontal regions (*p*s ≤ .001), but the amplitude difference between the left and right frontal regions was not significant (*p *= .1000). At baseline, there was no significant difference between the three scalp regions (*p*s = 1.000).

For the frontal LPP time window (400–750 ms), the mean amplitudes of directed remembering, directed forgetting, and baseline condition were .768, 1.696, and −.245 μV, respectively. The main effect of task type was significant, *F*(2, 48) = 8.636, *p* = .001, *η_p_*^2^ = .265, and the main effect of the frontal scalp region was significant, *F*(2, 48) = 9.318, *p* < .001, *η_p_*^2^ = .280. The task type × frontal scalp region interaction was significant, *F*(4, 96) = 4.699, *p* = .002, *η_p_*^2^ = .164. A simple effect test showed that the amplitude of the frontal LPP in the directed remembering condition was smaller than that in the directed forgetting condition (*p*s ≤ .011), regardless of the left-frontal, middle-frontal, or right-frontal regions. The directed forgetting condition of the three frontal scalp regions was significantly larger than that of the baseline condition (*p*s ≤ .018). A significant difference between the directed remembering and baseline conditions was found only in the right frontal region (*p *= .041) but not in the middle frontal and left frontal regions (*p*s ≥ .142). Both the directed remembering and directed forgetting conditions showed that the amplitude of the left frontal region was lower than that of the middle frontal and right frontal regions (*p*s ≤ .021), and there was no significant difference between the middle frontal and the right frontal regions (*p*s = 1.000), indicating that the frontal LPP was sourced from the right frontal region. There was no significant difference among the three scalp regions in the baseline condition (*p*s ≥ .715).

For the P3 time window (400–750 ms), the mean amplitudes of directed remembering, directed forgetting, and baseline conditions were 2.165, 2.543, and −.129 μV, respectively. A 3 (task type: directed remembering, directed forgetting, baseline) × 3 (parietal scalp regions: left parietal, middle parietal, right parietal) repeated-measures ANOVA was performed for P3. The main effect of task type was significant, *F*(2, 48) = 42.989, *p* < .001, *η_p_*^2^ = .642, and the main effect of parietal lobe region was significant, *F*(2, 48) = 10.422, *p* < .001, *η_p_*^2^ = .303. The interaction between task type and parietal scalp region was significant, *F*(4, 96) = 12.301, *p* < .001, *η_p_*^2^ = .339. The simple effect test showed no significant difference in P3 amplitude between the directed remembering and directed forgetting conditions in all parietal regions, *p*s ≥ .069. The P3 amplitude of the directed remembering condition was significantly larger than that of the baseline condition (*p*s ≤ .001), and the P3 of directed forgetting was also significantly larger than that of the baseline condition (*p*s ≤ .001). For the directed remembering condition, the P3 amplitude of the left parietal region was significantly lower than that of the middle and right parietal regions (*p*s ≥ .008), and there was no significant difference between the right and middle parietal regions (*p* = 1.000). For the directed forgetting condition, the P3 amplitudes of the left and right parietal regions were significantly lower than those of the middle parietal region (*p*s ≤ .049), and the amplitude of the left parietal region was significantly lower than that of the right parietal region (*p* = .050). For the baseline condition, there were no significant differences among the three parietal regions (*p*s = 1.000).

**Figure 3.**
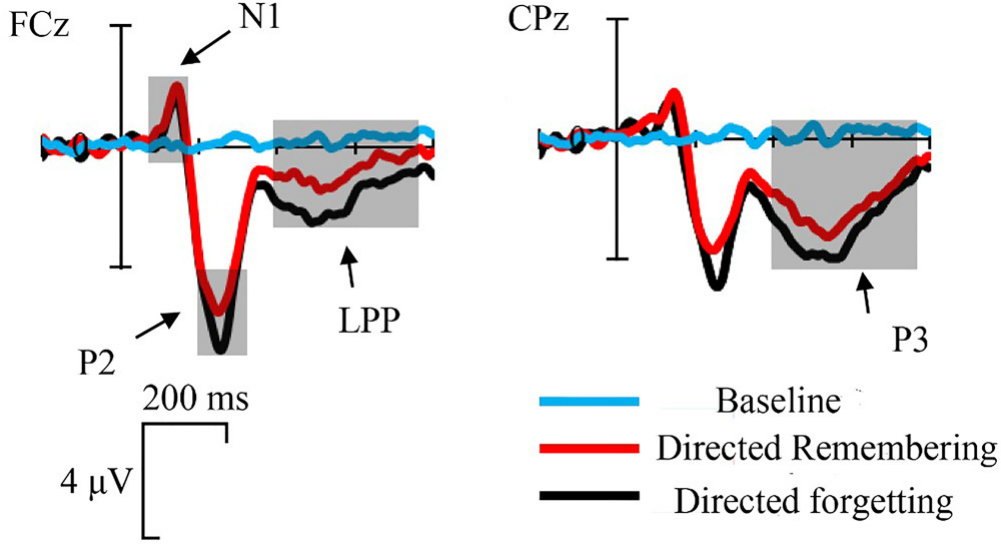
Grand averaged ERPs for the three conditions. FCz represents the electrophysiological activity of the frontal lobe, while CPz represents the electrophysiological activity of the parietal lobe. Results of ANOVAs revealed no significant difference in N1 induced by directed remembering and directed forgetting cue, but they were significantly larger than the baseline; Directed forgetting cue-induced the largest P2 amplitude, followed by directed remembering cue, and the baseline was the smallest. For frontal LPP, the amplitude induced by the directed forgetting cue is the largest, and there is no significant difference between the directed remembering cue and baseline. For P3, there was no significant difference in P3 induced by directed remembering and directed forgetting cues, but they were significantly larger than the baseline. The shadows represent time windows.

Abbreviations: ANOVA = analysis of variance; ERP = event-related potential; LPP = late positive potential.

**Figure 4.**
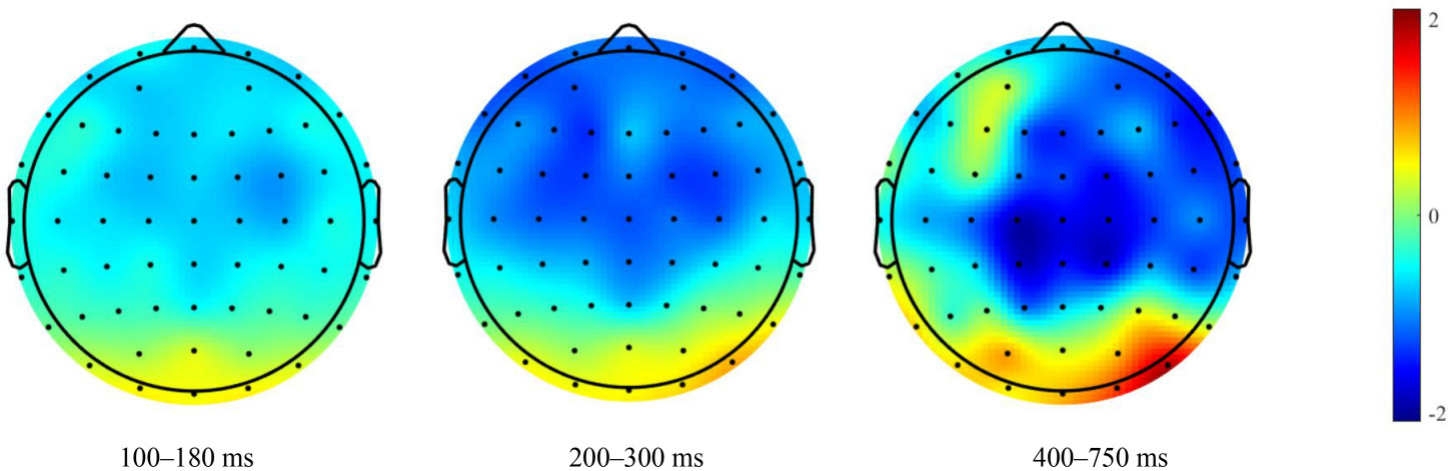
Topographical mapping of difference waves of directed remembering cue minus directed forgetting cue.

**Figure 5.**
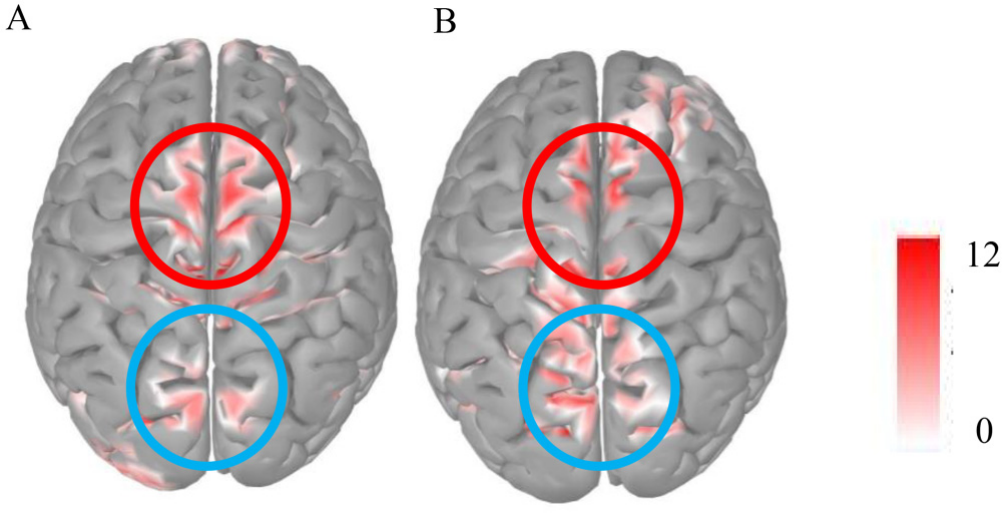
The source localization of directed remembering and directed forgetting condition during 400–750 ms. The red circle represents the frontal lobe, and the blue circle represents the parietal lobe.

To determine that frontal LPP and parietal P3 are two different ERP components in this study, the source localization was performed during 400–750 ms using the Brainstorm (Tadel et al., 2011) source analysis. Through source localization, the frontal and parietal lobes of the brain were activated simultaneously during 400–750 ms. Therefore, frontal LPP and parietal P3 were different ERP components localized from the frontal and parietal lobes, respectively (Figure 3 to 5).

### Correlational Analyses

**Figure 6.**
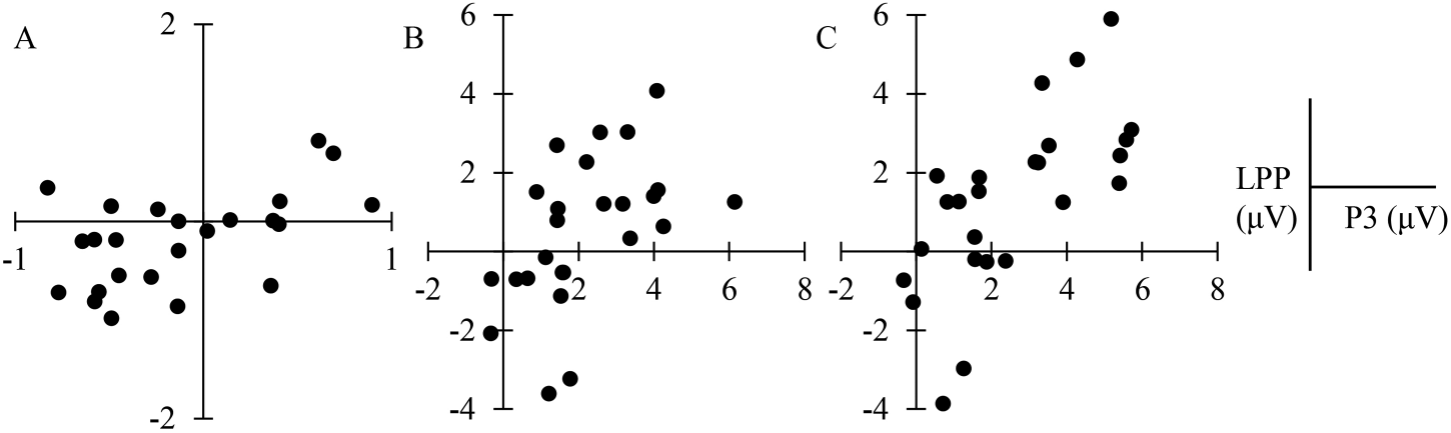
Correlation scatter chart. (A) The baseline condition with no significant correlation between P3 and frontal late positive potential (LPP); (B) the directed remembering condition with no significant correlation between P3 and frontal LPP; and (C) the directed forgetting condition, with a positive correlation between P3 and frontal LPP.

A partial correlation test was conducted between the P3 and frontal LPP amplitudes. After controlling the P2 amplitude for the directed remembering condition, no significant correlation was found between the P3 amplitude and frontal LPP amplitude (*r *= . 290, *p* = .169). However, a positive marginal correlation was observed for the directed forgetting condition between the P3 amplitude and frontal LPP amplitude (*r *= .399, *p* = .053, BF_01_ = .004). For the baseline condition, the correlation was not significant (*r *= .129, *p *= .549) (Figure 6).

To clarify the potential causality of the correlation between the EEG components of two different brain regions, we performed a Granger causality test. Eviews 11 was used for the Granger causality test. The unit root (stationary) for this study was tested by utilizing the augmented Dickey-Fuller (ADF). The ADF was performed for the LPP and P3 in the directed remembering and directed forgetting condition, respectively, and the results revealed that they were both significant (*p*s ≤ .002). Therefore, we conducted Granger causality tests for LPP and P3 in the directed remembering and directed forgetting conditions, respectively, with the lag set to 4. For the directed remembering condition, the Granger causality for both frontal LPP to parietal P3 and parietal P3 to frontal LPP were not significant (*p*s ≥ .222). For the directed forgetting condition, the Granger causality for parietal P3 to frontal LPP was not significant (*p* = .236), but frontal LPP to parietal P3 was significant (*p* = .005).

## Discussion

This study explored the presence of active inhibitory components in RCE. Williams and Woodman (2012) found that accuracy was significantly higher for the directed remembering and directed forgetting conditions than for the baseline condition and significantly higher for the directed remembering condition than for the directed forgetting condition. However, this study did not find such a pattern in accuracy results, which may be due to the sufficient interval between the memory and test stages (2800–3200 ms); thus, the visual working memory content was consolidated (Long et al., 2019; Sun & Fu, 2011). However, RT results showed that RTs for the directed remembering and directed forgetting conditions were significantly lower than those for the baseline condition, and RTs for the directed remembering condition were significantly lower than those for the directed forgetting condition, similar to the results of some studies (Lin et al., 2021; Xu & Tanaka, 2013). The result of RT may reflect that after the visual working memory content is consolidated, the retro-cue can also play a role and optimize task performance, improving the speed of memory information extraction in visual working memory. This study found that after the visual working memory content is consolidated, retro-cue improves the speed of extracting information in visual working memory. To support this result, we calculated BIS indicators based on the recommendations by Liesefeld and Janczyk (2022) and found no significant differences in BIS indicators for all three conditions. Combining the ACC and RT results, these BIS results suggest that the cue did not result in better overall task performance when the memory interval was extended, but only sped up the RT.

At the cue stage, this study found no significant difference between the two cue conditions in the N1 component, which is closely related to early sensory perception (Sponheim et al., 2006; Sun et al., 2012), reflecting the degree of the individual attention alert state (Qi & Gao, 2020; Shackman et al., 2011). In this study, the two cues evoked N1 with no significant difference, which not only reflected that the two cues triggered the participants’ attention and vigilance to the same extent but also explained the rationality of setting directed remembering and directed forgetting cues as solid and hollow words, respectively. Therefore, the differences in subsequent electrophysiological responses are not caused by the physical properties of cue stimuli but by different patterns of internal attention distribution triggered by cues. In the subsequent time window, directed remembering cues induced a smaller P2 than those of directed forgetting cues. Many studies have indicated that P2 is closely related to selective attention before cognitive control (Bergstrom et al., 2007; Lenartowicz et al., 2014; Mecklinger et al., 2009). The present study found that the P2 amplitude in the directed forgetting condition was significantly larger than that in the directed remembering condition, which may indicate that participants gave more attentional resources in the directed forgetting task. These attentional resources may be used for continuous inhibition of task-irrelevant information induced by the directed forgetting cue, which is reflected in the amplitude of the frontal LPP.

The hippocampus is closely associated with memory formation (Squire, 1992). The higher the activation of the hippocampus, the higher the memory intensity (Davachi et al., 2003; Fernández et al., 1999). Memory inhibition requires individuals to stop the process of repetition or retrieval through active inhibition. The DLPFC plays an important role in many tasks, such as the inhibition of dominant behavioral responses (Aron et al., 2004; Garavan et al., 2002; Menon et al., 2001), task switching (Nakahara et al., 2002), and cognitive inhibition (Bunge et al., 2001; Jonides et al., 1998). Anderson et al. (2004) pointed out that individuals can inhibit memory of task-irrelevant information by enhancing the activity of DLPFC to weaken hippocampal activity. Studies by Gagnepain et al. (2017) and Depue et al. (2016) also revealed that the higher the activation of DLPFC, the lower the activation level of the hippocampus. Specifically, DLPFC can regulate the activity of the hippocampus and weaken memory strength by inhibiting the activity of the hippocampus. Studies found that the frontal LPP amplitude was more positive for memory inhibition than memory retrieval conditions (Cano & Knight, 2016; Mecklinger et al., 2009). Ma et al. (2020) asserted that the frontal LPP reflected the activity of the DLPFC, representing the enhancement of DLPFC activity aimed at weakening hippocampal activity. The results of this study exhibited that compared with the directed remembering cue and the baseline, the frontal LPP amplitude induced by the directed forgetting cue was more positive, while the frontal lobe LPP amplitudes induced by the baseline and the directional remembering cue were not significantly different. These results may indicate that the directed forgetting cue makes individuals make a more cognitive effort to continuously inhibit task-irrelevant information, while individuals did not make cognitive efforts to continuously inhibit task-irrelevant information for the baseline and directed remembering cues. At the same time, this study found that the amplitude of LPP in the right frontal region was the largest among the three conditions, similar to Ma et al. (2020). Depue et al. (2016) and Garavan et al. (1999) also suggested that the right DLPFC plays an important role in memory inhibition, thereby regulating hippocampal activity. Therefore, in this study, the enhancement of frontal LPP (especially in the right frontal region) induced by directed forgetting cues may reflect the continuous active inhibition control process of memory when task-irrelevant information intrudes into the memory.

Traditional forgetting studies (e.g., directed forgetting [Gao et al., 2019]) adopted N2 as a neural marker of inhibition but did not discuss frontal LPP. This may be due to the differences in experimental paradigms: in the traditional directed forgetting paradigm, participants only needed to inhibit the word when it was presented and then entered the next attempt without continuous inhibition. However, the retro-cue paradigm used in this study presents the test stage immediately at the end of a trial. Hence, the participants needed to continuously inhibit task-irrelevant information to prevent it from interfering with the repetition of task-irrelevant information. In addition, the number of items in this study that participants need to continuously inhibit is three, which requires more inhibition efforts (Feldmann-Wüstefeld & Vogel, 2019). This also allowed us to observe the continuous memory inhibition process of the frontal LPP response.

This study found no significant difference in P3 amplitude induced by directed remembering and directed forgetting cues. P3 is closely related to memory encoding (Azizian & Polich, 2007; Gilmore et al., 2018), and memory strategies controlled by consciousness are closely related to P3 amplitude (Christie et al., 2012). According to Gao et al. (2016a, 2016b) and Patrick et al. (2015), P3 may be a neural marker for selective repetition P3 may be closely related to the selective retelling and encoding of information. The more cognitive resources retelling information occupies, the larger the P3 amplitude that would be induced. Thus, the results of this study may indicate that the cognitive resources used for memory retrieval are the same in the directed remembering and directed forgetting conditions. In addition, a traditional directed forgetting study found that the P3 amplitude was correlated with the behavioral correct recall rate of memory information (Patrick et al., 2015; Pierguidi et al., 2016; Yang et al., 2012). Similarly, in this study, although the participants were required to manipulate the items in visual working memory according to two different cues, the task-relevant items under the two cues had the same level of retelling processing evidence based on the results of P3 and behavioral recall accuracy. Compared with the baseline condition, the P3 amplitudes of both the directed remembering and directed forgetting conditions were significantly larger. This may be because the baseline condition did not provide a cue to guide participants in conducting cognitive operations. The participants coded and consolidated the memory items from the memory stage to the test stage. Therefore, no memory strategies guided by cues were used in the baseline condition. Combining the results of the frontal LPP and P3, for the directed remembering condition, the intensity of task-irrelevant information representation was passively reduced, while for the directed forgetting condition, the intensity of task-irrelevant information representation was actively suppressed. This finding is similar to that of Williams and Woodman (2012). They found that participants guided by directed remembering cues only needed to repeat task-relevant information, while participants guided by directed forgetting cues not only required to repeat task-relevant information but also inhibit task-irrelevant information.

Based on the high temporal resolution of ERP, we observed that different cues induced different cognitive activities. Specifically, when the cue was directed toward remembering, the participants deployed attention resources to task-relevant information and intensively retold them. However, when the cue was directed toward forgetting, the participants needed to recruit more attentional resources. On the one hand, they need to strengthen the retelling of task-relevant information. On the other hand, they need to inhibit task-irrelevant information. It should be noted that compared with the directed remembering condition, in which participants only needed to enhance task-irrelevant information, participants in the directed forgetting condition needed to perform two different cognitive operations (inhibition and enhancement), which may lead to the prolongation of participants’ behavioral reaction time.

The boost and bounce theory proposed by Olivers and Meeter (2008) propose that working memory is a workspace that controls the overall situation, and the information stored in it includes both task-relevant and task-irrelevant information. Task-relevant information will be enhanced by excitatory operations, while task-irrelevant information will be weakened by inhibitory operations. Poch et al. (2018) and Schneider et al. (2019) also proved that working memory needs to inhibit task-irrelevant information and enhance task-relevant information. Fu et al. (2020) pointed out that whether the two processing methods, enhancement, and inhibition, affect each other has not been proven experimentally. To investigate whether enhancement of task-relevant information and inhibition of task-irrelevant information interact or do not interfere, the present study conducted a partial correlation analysis between the repetitive neural marker P3, which reflects enhancement of task-relevant information, and the frontal LPP, which reflects sustained inhibition of task-irrelevant information. P2 may reflect the amount of attentional resources recruited (Bergstromen et al., 2007; Lenartowicz et al., 2014; Mecklinger et al., 2009). These attentional resources are used to inhibit task-irrelevant information or enhance task-relevant information, which is the preparation state of participants for the inhibition or enhancement process. Therefore, when investigating the relationship between the inhibition or enhancement processes, the effect of P2 should be excluded.

After controlling for the P2 amplitude, we found no significant correlation between P3 and frontal LPP for directed remembering and baseline conditions, which may be due to the active inhibition of task-irrelevant information that was not induced in these two conditions. However, for the directed forgetting condition, P3 was positively correlated with LPP in the frontal lobe, and Bayesian analysis tended to be significantly correlated, which may indicate that there is a potential relationship between active inhibition and enhancement. Furthermore, the Granger cause test results indicated that the frontal LPP is Granger causal to the parietal P3 in the directed forgetting condition. It has been found that visual information is processed in the occipital lobe (Tong, 2003), while the prefrontal cortex provides inhibitory control (Miller & Cohen, 2001). The fronto-parietal network model postulates that when the visual cortex processes visual information and finds task-irrelevant information, it activates the frontal cortex to inhibit task-irrelevant information (Zimmer, 2008). The results of this study conform to the fronto-parietal network model and further explain that after individuals inhibit task-irrelevant information, cognitive resources stripped from task-irrelevant information may be reallocated to task-relevant information to enhance their memory intensity. This result also exhibits that the continuous resource model is more likely to explain the RCE caused by a directed remembering cue, while the boost and bounce theory is more likely to explain the RCE caused by a directed forgetting cue.

A limitation of this study is that it only solves the problem of whether active inhibition components are present in RCE and does not discuss how active inhibition changes with load. Ergen et al. (2012) found that different memory loads had different inhibition strengths. Based on this, it may be possible to explore the changes in active inhibition components in RCE with load by setting the working memory information for different loads.

### Conclusions

There is an active inhibitory component in the RCE of visual working memory that manifests as a frontal LPP component induced by a directed forgetting cue. There is a potential correlation between the enhancement of task-relevant information and the weakening of task-irrelevant information in the directed forgetting condition.
